# Increased IGFBP7 Expression Correlates with Poor Prognosis and Immune Infiltration in Gastric Cancer

**DOI:** 10.7150/jca.50370

**Published:** 2021-01-01

**Authors:** Qiaoyun Zhao, Rulin Zhao, Conghua Song, Huan Wang, Jianfang Rong, Fangfei Wang, Lili Yan, Yanping Song, Yong Xie

**Affiliations:** 1 Department of Gastroenterology, The First Affiliated Hospital of Nanchang University, No.17, Yongwai Zheng Street, Donghu District, Nanchang, 330000, Jiangxi, China.; 2 Laboratory of Biochemistry and Molecular Biology, Jiangxi Institute of Medical Sciences, Nanchang 330000, Jiangxi Province, China.

**Keywords:** IGFBP7, prognosis, immune infiltration, gastric cancer, bioinformatics analysis

## Abstract

**Background:** Insulin-like growth factor binding protein-7 (IGFBP7) contributes to multiple biological processes in various tumors. However, the role of IGFBP7 in gastric cancer (GC) is still undetermined. The study aims to explore the role of IGFBP7 in GC via an integrated bioinformatics analysis.

**Methods:** IGFBP7 expression levels in GC and its normal gastric tissues were analyzed using multiple databases, including the Tumor Immune Estimation Resource (TIMER), Oncomine, The Cancer Genome Atlas (TCGA) and Gene Expression Omnibus (GEO) databases, as well as by our clinical gastric specimens. The methylation analysis was conducted with MEXPRESS, UALCAN and Xena online tools. The survival analysis was conducted using the Kaplan-Meier Plotter and Gene Expression Profiling Interactive Analysis (GEPIA) databases. Coexpressed genes of IGFBP7 were selected with the cBioPortal tool and enrichment analysis was conducted with the clusterProfiler package in R software. Gene set enrichment analysis (GSEA) was performed to explore the IGFBP7-related biological processes involved in GC. Correlations between IGFBP7 and immune cell infiltrates were analyzed using the TIMER database.

**Results:** IGFBP7 expression was significantly upregulated in GC and correlated with stage, grade, tumor status and *Helicobacter pylori* infection. High IGFBP7 expression and low IGFBP7 methylation levels were significantly associated with short survival of patients with GC. Univariate and multivariate analyses revealed that IGFBP7 was an independent risk factor for GC. The coexpressed genes *LHFPL6*, *SEPTIN4*, *HSPB2*, *LAYN* and* GGT5* predicted unfavorable outcomes of GC. Enrichment analysis showed that the coexpressed genes were involved in extracellular matrix (ECM)-related processes. GSEA indicated that IGFBP7 was positively related to ECM and inflammation-related pathways. TIMER analysis indicated that the mRNA level of IGFBP7 was strongly correlated with genes related to various infiltrating immune cells in GC, especially with gene markers of tumor associated macrophages (TAMs).

**Conclusions:** Increased IGFBP7 expression correlates with poor prognosis and immune cell infiltration in GC, which might be a potential biomarker for the diagnosis of GC.

## Introduction

Gastric cancer (GC) remains an important malignant tumor with more than one million new cases and 783,000 deaths globally in 2018 1. *Helicobacter pylori* (*H. pylori*) is the major risk factor for GC and causes chronic gastric inflammation and actives cancer-related signaling pathways 2. In the past few decades, as significant progress has been made in the early diagnosis and treatment of GC, and *H. pylori* infection has been effectively eradicated on a large scale, the incidence and mortality of GC have dropped considerably 3, 4. However, the prognosis of GC remains poor. Aberrantly expressed genes in GC may participate in tumorgenesis and cancer progression, which are related to unfavorable outcomes of GC. Therefore, identification of new potential biomarkers for the diagnosis and targeted therapy of GC is urgently needed.

Insulin-like growth factor (IGF) is well known to be involved in cell growth and regulated by a group of IGF binding proteins (IGFBPs) and their receptors 5. Insulin-like growth factor binding protein-7 (IGFBP7) is a secreted protein that binds to the IGF-I receptor with low affinity and blocks insulin action 6, 7. IGFBP7 acts as an IGF-dependent and IGF-independent cell growth regulator. Its IGF-independent actions seem to contribute to tumor cell growth, angiogenesis, epithelial mesenchymal transition (EMT) and cell apoptosis 8-13. The expression level of IGFBP7 is regulated by DNA methylation in various cancer types 14-17. IGFBP7 is assumed to be a growth suppressing gene in some tumors, because reduced expression has often been found in these tumors, such as in colorectal cancer, hepatocellular cancer, and thyroid cancer 18-21. However, it has been reported that IGFBP7 levels in serum in the esophageal squarmous cell cancer are significantly higher than those in normal tissues and might be a potential biomarker for diagnosis 22. Another study utilizing mass spectrometry-based proteomics methods revealed that IGFBP7 was overexpressed in head and neck squamous cell carcinomas, which was associated with inferior survival and an increased risk of disease progression 23.

In gastric cancer, the role of IGFBP7 is undetermined 24, 25. Therefore, studies with substantial sample sizes and utilizing omic technologies are needed to confirm the role of IGFBP7 in GC. In this study, we performed a comprehensive analysis of IGFBP7 expression and epigenetic regulation and its correlation with clinical outcomes in GC patients using multiple available databases and our clinical specimens. To investigate the possible pathogenic mechanism of IGFBP7 in GC, we further performed enrichment analysis of coexpressed genes and gene set enrichment analysis (GSEA) of IGFBP7. Finally, the relationship between IGFBP7 and immune cell infiltration was also explored. Our work highlights the important role of IGFBP7 on the carcinogenicity and prognosis in GC and indicates that IGFBP7 could act as a potential therapeutic target for GC.

## Materials and Methods

### Expression analyses of IGFBP7

Tumor Immune Estimation Resource (TIMER, https://cistrome.shinyapps.io/timer/) is a comprehensive tool for analyzing immune infiltrates according to diverse cancer types 26, and was first used to analyze the mRNA levels of IGFBP7 in 33 cancers based on TCGA expression profile data. The Oncomine database (https://www.oncomine.org/resource/login.html) is an accessible online cancer microarray database that was further used to explore the mRNA expression levels of IGFBP7 in different subtypes of GC with log2-transformed expression data. The filter thresholds used in the Oncomine database analysis were defined as follows: *P*-value < 1E-4, fold change >2 and gene rank in the top 10%.

The expression and phenotype data of the TCGA-STAD dataset were obtained from the GDC Hub of UCSC Cancer Genome Browser (http://xena.ucsc.edu/). For gene expression, HTSeq-count data were retrieved. The expression profiles of GSE54129, GSE15459, GSE79973 and GSE118916 were downloaded from the Gene Expression Omnibus (GEO) database (https://www.ncbi.nlm.nih.gov/geo/) and were listed in Table S1. The expression matrix was log2 transformed in R (version 3.6.1) if necessary.

### Gastric tissue specimens and immunohistochemistry

Sixteen pairs of paraffin-embedded human GC and adjacent normal gastric tissues were collected from surgical samples in the first affiliated Hospital of Nanchang University. None of patients were treated with adjuvant chemotherapy. All patients signed the informed consents and this study was approved by the Ethics Committee of the First Affiliated Hospital of Nanchang University. Immunohistochemical staining of IGFBP7 (cat#ab74169, Abcam, Cambridge, UK) was performed at a concentration of 1:800 and was scored independently by two pathologists.

### RNA extraction and real-time fluorescence quantitative PCR analysis

Total RNA was extracted from 16 paired GC and normal tissues using Trizol Regents (Takara, Dalian, China) and was reverse transcribed into cDNA using PrimeScript RT regent kit (Takara, Dalian, China) according to the manufacturer's instructions. The mRNA levels of IGFBP7 was measured by using SYBR Green Master Mix (Thermo Fisher Scientific) in the QuantStudio^TM^ 5 Real-Time PCR system (Thermo Fisher Scientific). The endogenous β-actin mNRA level was used to normalize the IGFBP7 transcript levels by using the 2^-ΔΔCT^ method.

### Methylation analyses of IGFBP7

The MEXPRESS tool (https://mexpress.be/index.html) was used to visualize the expression, methylation and clinical data from TCGA 27, 28, which was used to visualize the mRNA expression and methylation of IGFBP7 in GC. Promoter methylation levels of IGFBP7 in GC and its normal gastric tissues were analyzed in UALCAN online tool (http://ualcan.path.uab.edu/cgi-bin/ualcan-res.pl). The overall survival analysis of IGFBP7 methylation was conducted in UCSC Cancer Genome Browser (http://xena.ucsc.edu/).

### Survival analyses of IGFBP7

The Kaplan-Meier Plotter tool (https://kmplot.com/analysis/) was used to assess the effect of IGFBP7 expression on survival in GC with microarray data from the GEO database. The query probe ID 213910_at was examined. A total of 875 GC samples, 640 GC samples and 498 GC samples were used to analyze the correlation between IGFBP7 expression and overall survival (OS), first progression survival (FPS) and post progression survival (PPS), respectively. In addition, Gene Expression Profiling and Interactive Analysis (GEPIA, http://gepia.cancer-pku.cn/detail.php) was further used to verify the survival analysis based on RNA-seq data from the TCGA database. The median expression value of IGFBP7 was used to divide GC samples into high and low groups. The log-rank method was used for the hypothesis test. A *P*-value <0.05 was considered statistically significant.

### Analyses of genes coexpressed with IGFBP7

First, the top eight coexpressed genes of IGFBP7 according to adjusted *P* values were selected, and correlation analysis was performed in the cBioPortal online database (https://www.cbioportal.org/). Second, survival analysis of the eight genes was conducted using the GEPIA online tool. Finally, the top 200 coexpressed genes of IGFBP7 were selected, and enrichment analysis was performed with the clusterProfiler package in R (version 3.6.1).

### Gene set enrichment analysis (GSEA)

To explore the potential pathogenic biological processes of IGFBP7, we conducted GSEA for TCGA-STAD data using GSEA v4.3.0 software. The gene set c2.cp.kegg.v7.1.symbols.gmt was selected for further analysis. The number of permutations was 1000. NES (normalized enrichment score) >1 and FDR FDR (false discovery rate) q-val <0.05 were set as cut-offs for significant enrichment.

### Immune infiltration analysis

Correlations between IGFBP7 expression and various infiltrating immune cell types were investigated using the TIMER database. Additionally, correlations between IGFBP7 expression and gene markers of infiltrating immune cell types were also explored.

### Statistical analyses

Scatter plots were generated using GraphPad Prism 7.0. SPSS 21.0 software was used to conduct statistical analyses. A standard Student's t-test, paired t-test or Mann-Whitney U-test was used to compare the difference within two groups. ANOVA analysis or Kruuskal-Wallis test was used to compare difference among more than two groups. Univariate and multivariate Cox regression analyses were conducted in R. *P*-value <0.05 was considered statistically significant.

## Results

### Transcriptional and methylation levels of IGFBP7 in patients with GC

**Figure 1:**
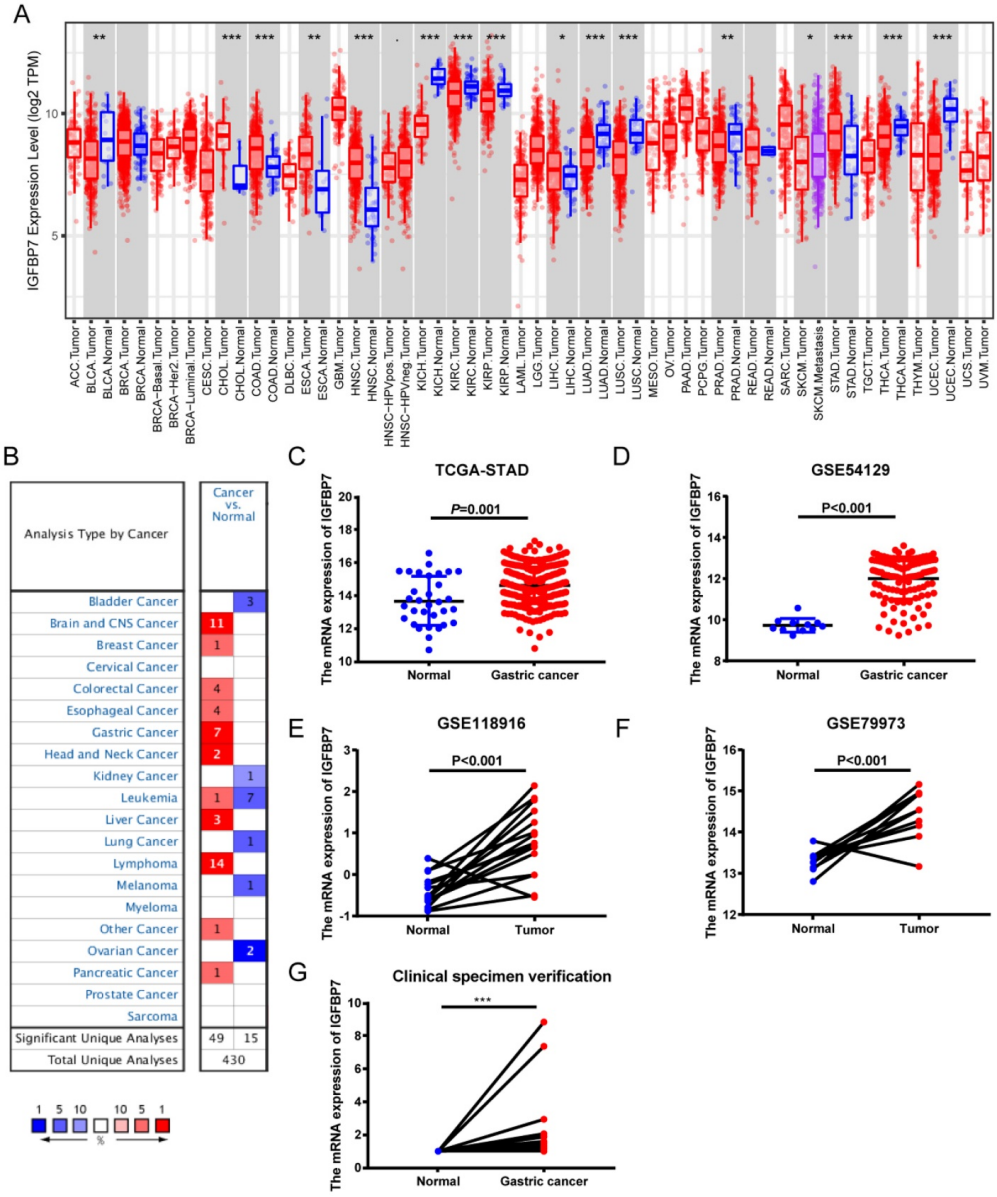
The transcription level of IGFBP7 was increased in gastric cancer. A, The expression of IGFBP7 in different types of cancers as assessed with the TIMER database. *, *P*<0.05; **, P<0.01; ***, *P*<0.001. B, The expression of IGFBP7 in different types of cancers as assessed with the Oncomine database. Colors: red, high expression; blue, low expression. C-F, The expression of IGFBP7 in gastric cancer based on TCGA (C), GSE54129 (D), GSE118916 (E), and GSE79973 (F) datasets. G, the mRNA levels in 16 paired GC and normal gastric tissues collected from our hospital.

To determine the expression levels of IGFBP7 in different cancers, we analyzed IGFBP7 expression among various cancers using the TIMER database. The results revealed that the expression levels of IGFBP7 were divergent in different cancers. In stomach adenocarcinoma (STAD), the expression of IGFBP7 was significantly increased compared to that in normal tissues (Figure 1A). Oncomine database further revealed that IGFBP7 mRNA expression was upregulated in GC among seven datasets (Figure 1B). Moreover, increased IGFBP7 expression in GC was confirmed using TCGA (Figure 1C) and three GEO datasets (Figure 1D-F). In addition, the increased mRNA levels of IGFBP7 in GC were also confirmed in 16 paired GC and normal gastric tissues collected from our hospital (Figure 1G).

Furthermore, the IGFBP7 mRNA levels in Lauren type of gastric cancer were also explored. As we can see, the mRNA expression of IGFBP7 was significantly upregulated in gastric diffuse adenocarcinoma, intestinal type adenocarcinoma, and mixed adenocarcinoma compared to normal gastric tissues (Table 1). Taken together, the results of multiple datasets suggested that the mRNA expression of IGFBP7 was significantly upregulated in GC tissues compared to normal gastric tissues.

To examine whether the mRNA expression of IGFBP7 was regulated by DNA methylation, we used the MEXPRESS tool to visualize the gene expression and methylation levels of IGFBP7. The results revealed that all probes in the promoter region showed a significantly negative correlation with IGFBP7 mRNA levels (Figure S1A). In addition, the promoter methylation level of IGFBP7 was reduced in GC compared to that in normal gastric tissue (Figure S1B), and promoter hypomethylation was associated with a poor prognosis of GC (Figure S1C). Altogether, the results showed that the expression of IGFBP7 was negatively regulated by methylation and was associated with prognosis of GC.

### IGFBP7 protein was upregulated in clinical GC specimens

**Figure 2:**
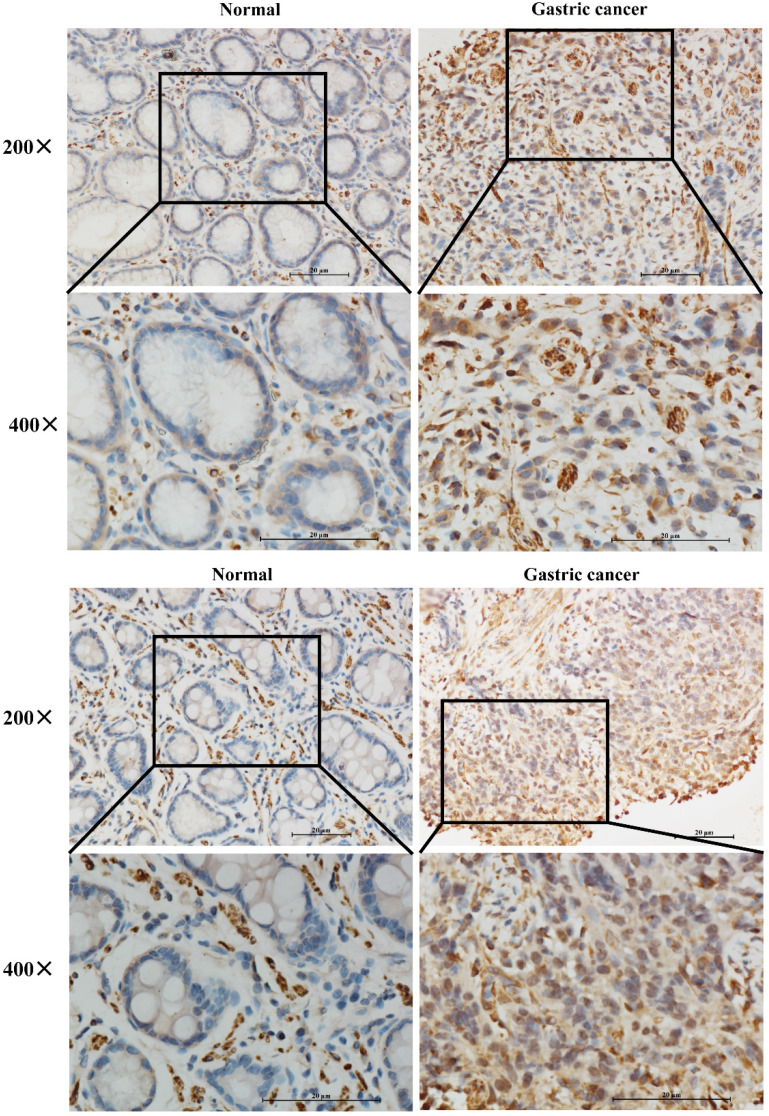
Expression levels of IGFBP7 in paraffin-embedded GC and normal gastric tissues.

To further verify whether IGFBP7 was higher expressed in GC patients, we performed IHC staining of IGFBP7 in 16 paired GC and normal tissues. The results showed that IGFBP7 was significantly higher expressed in GC specimens than that of in normal tissues (Figure 2).

### Relationship between IGFBP7 and clinicopathological characteristics of GC patients

**Figure 3:**
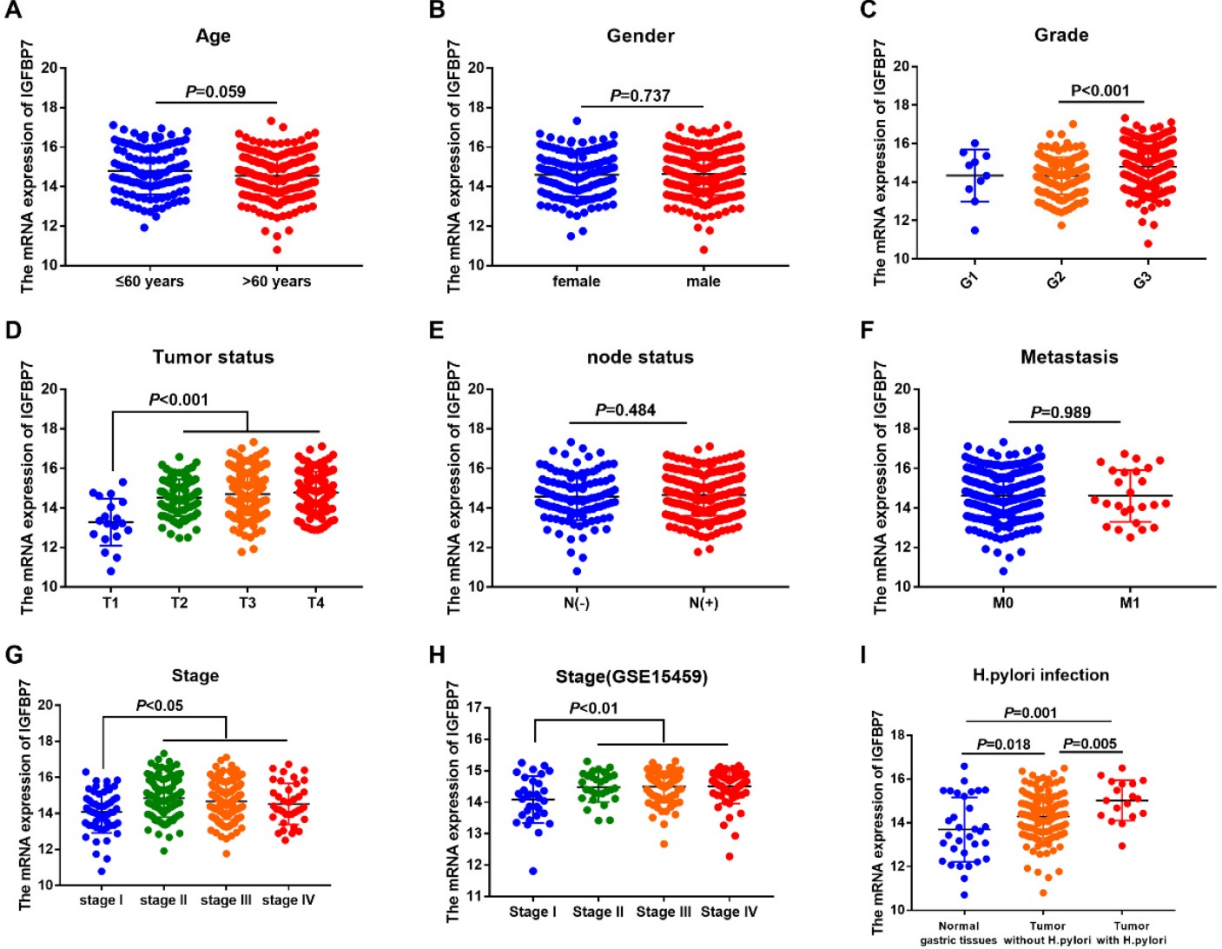
Association between IGFBP7 expression and clinicpathological characteristic of GC patients. The mRNA expression level of IGFBP7 is shown with scatter plots using the TCGA-STAD dataset according to age (A), sex (B), grade (C),tumor status (D), node status (E), metastasis (F), stage (G) and *H. pylori* infection (I), as well as according to stage in GSE15459 dataset (H).

To explore the relationship between the mRNA expression of IGFBP7 and the clinicopathological characteristics of GC patients, we analyzed clinical information of GC samples from the TCGA-STAD project. The results revealed that the mRNA expression of IGFBP7 was significantly increased in the G3 phase (*P*<0.001, Figure 3C), advanced tumor status (T2/3/4) (*P*<0.001, Figure 3D) and advanced stages (II/III/IV) (*P*<0.05, Figure 3G). The correlation between IGFBP7 expression and stage was also confirmed using the GSE15459 dataset (*P*<0.01, Figure 3H). The expression level of IGFBP7 was significantly higher in GC samples with *H. pylori* infection than that in GC samples without *H. pylori* infection (*P*=0.001, Figure 3I). However, the mRNA expression of IGFBP7 showed no significant correlation with age, gender, node status and metastasis status (*P*>0.05, Figure 3A, B, E, F).

### High IGFBP7 expression predicted a poor prognosis in GC patients

**Figure 4:**
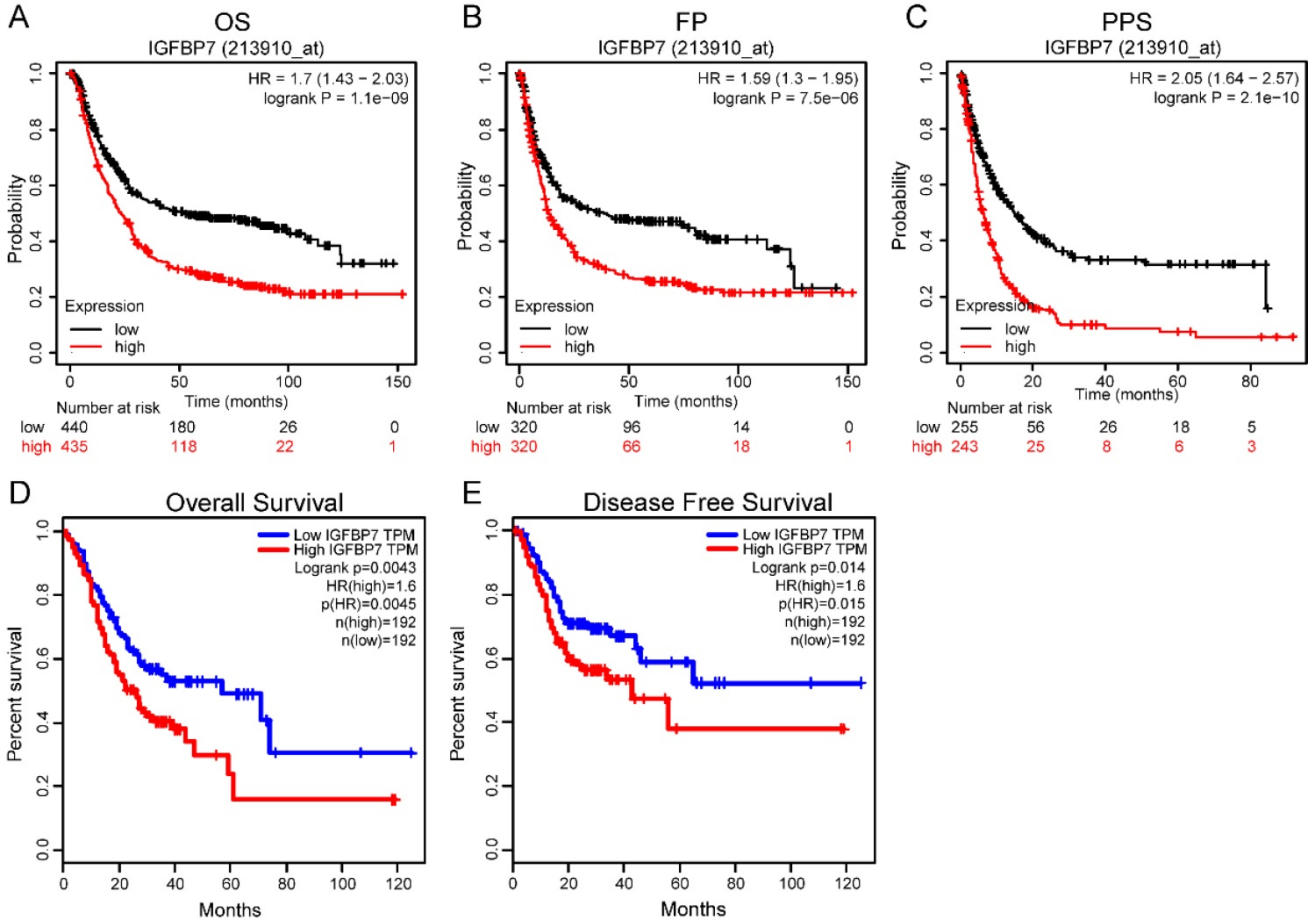
High IGFBP7 expression was associated with poor prognosis in GC. A-C, The relationship of IGFBP7 mRNA expression with overall survival (OS) (A), first progression survival (FPS) (B) and post progression survival (PPS) (C) was assessed with probe 213910_at in the Kaplan-Meier Plotter database. D-E, The association of IGFBP7 mRNA expression and overall survival (D) and disease-free survival (E) was assessed in the GEPIA database. The median value was used as the cutoff for dividing the high and low groups.

To explore the prognostic value of IGFBP7 in GC patients, we used GC sample data based on microarray chip and transcriptome sequencing from two different databases. The microarray chip results revealed that high IGFBP7 expression was strongly associated with poor overall survival (OS, Figure 4A), first progression survival (FP, Figure 4B) and post progression survival (PPS, Figure 4C) of GC. Furthermore, the analysis based on transcriptome sequencing data indicated that high IGFBP7 expression was significantly related to poor overall survival (Figure 4D) and disease-free survival (Figure 4E).

In addition, univariate and multivariate Cox regression analyses revealed that high IGFBP7 expression was an independent risk factor for unfavorable survival of GC (Table 2).

### Analyses of genes coexpressed with IGFBP7 in GC

**Figure 5:**
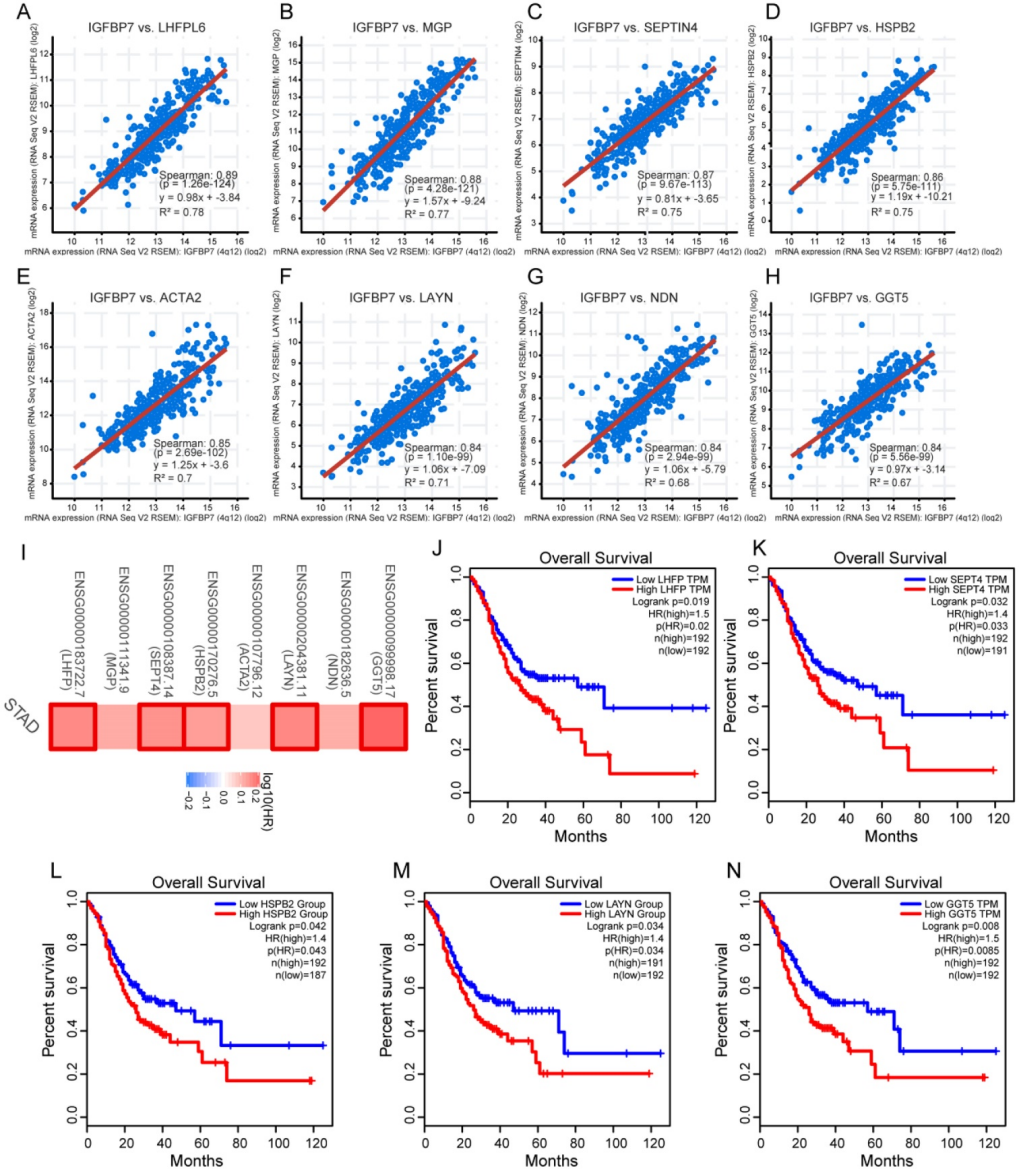
Analyses of genes coexpressed with IGFBP7. A-H, The top eight coexpressed genes of IGFBP7 were selected and correlation analysis was performed in the cBioPortal online database. These genes included LHFPL6 (A), MGP (B), SEPT4 (C), HSPB2 (D), ACTA2 (E), LAYN (F), NDN (G) and GGT5 (H). I, Survival map of the 8 coexpressed genes based on TCGA-STAD project data was generated with the GEPIA online tool. A red box indicates those genes with statistical significance. J-N, Overall survival analysis was performed for LHFPL6 (J), SEPT4 (K), HSPB2 (L), LAYN (M) and GGT5 (N). Briefly, GC samples were divided into high and low expression groups by the median expression value of the gene. The log-rank method was used for the hypothesis test.

Coexpressed genes typically have similar functions. The top eight coexpressed genes of IGFBP7 arranged by adjusted *P* values were identified. The correlation analysis revealed that IGFBP7 was highly positively correlated with tetraspanin subfamily member 6 (*LHFPL6*), matrix Gla protein (*MGP*), septin 4 (*SEPTIN4*), heat shock protein family B (Small) member 2 (*HSPB2*), actin alpha 2, smooth muscle (*ACTA2*), layilin (*LAYN*), necdin, MAGE family member (*NDN*) and gamma-glutamyltransferase 5 (*GGT5*) (Figure 5A-H). The survival map indicated that all 8 genes were risk factors for unfavorable survival of GC, among which five genes showed statistically significant relationships with survival (Figure 5I). Overall survival analysis further confirmed that high expression levels of *LHFPL6*, *SEPTIN4*, *HSPB2*, *LAYN* and *GGT5* were associated with poor prognosis of GC (Figure 5J-N).

To explore the potential biological function of genes coexpressed with IGFBP7, top 200 genes were selected to conduct the enrichment analysis. The GO biological process (BP) analysis showed that the terms extracellular structure organization, extracellular matrix organization, muscle system process, ameboidal-type cell migration and regulation of cellular response to growth factor stimulus were significantly enriched (Figure S2A). The GO term cellular component (CC) analysis showed that the terms collagen-containing extracellular matrix, contractile fiber and extracellular matrix component were significantly enriched (Figure S2B). The molecular function (MF) analysis showed that the terms extracellular matrix structural constituent, glycosaminoglycan binding and collagen binding were mainly enriched (Figure S2C). KEGG pathway analysis showed that vascular smooth muscle contraction was significantly enriched (Figure S2D). Overall, the enrichment analysis indicated that IGFBP7 and its coexpressed genes may be involved in extracellular matrix- related signaling processes.

### GSEA identified IGFBP7-related pathways

**Figure 6:**
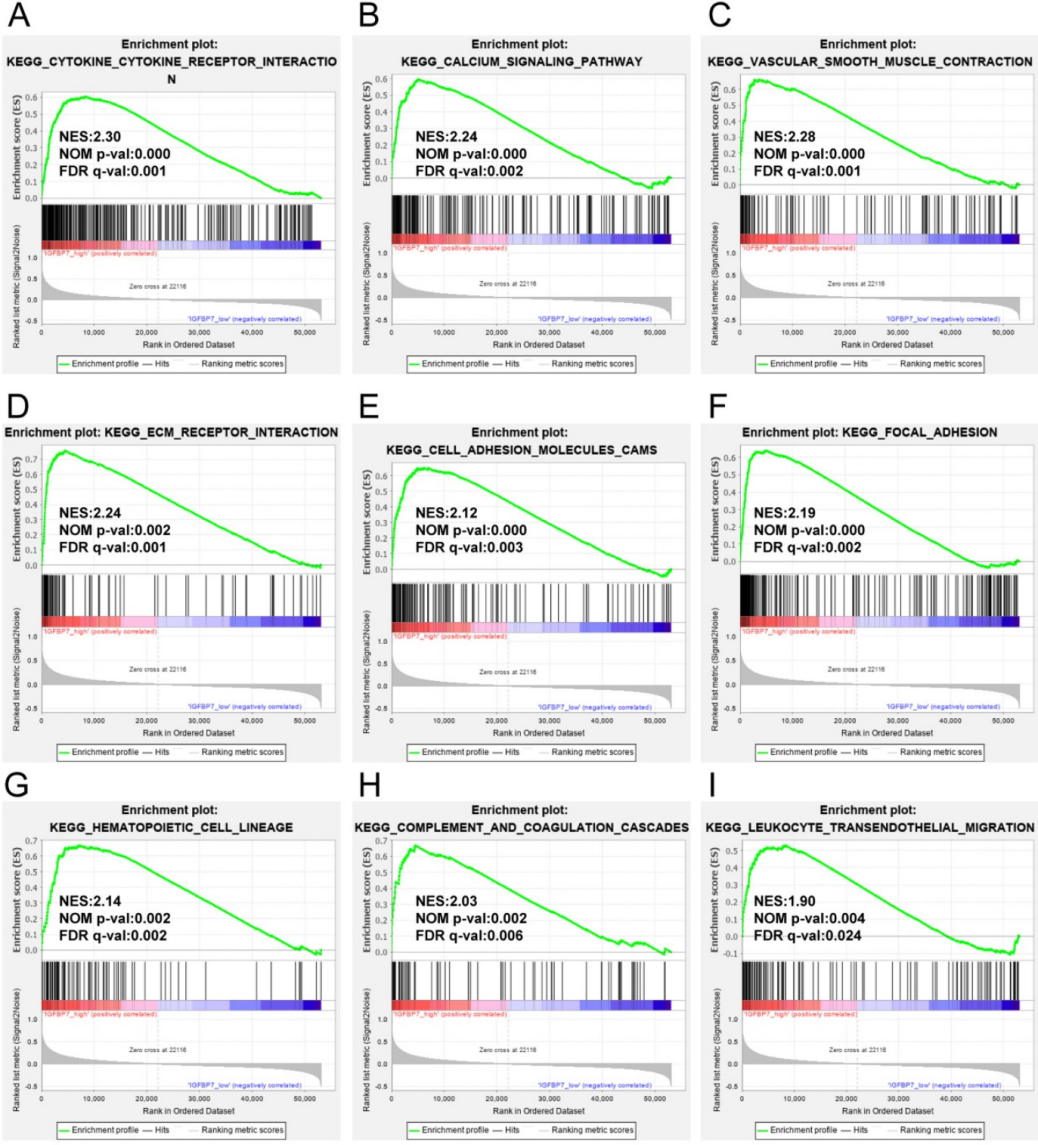
GSEA analysis of IGFBP7 based on expression in the TCGA-STAD dataset. NES: normalized enrichment score; NOM p-value: nominal p value; FDR q-val: false discovery rate.

To analyze the possible biological pathways regulated by IGFBP7 in GC, we conducted GSEA between high and low IGFBP7 expression groups based on the TCGA-STAD dataset. A total of 22 pathways were significantly enriched in the IGFBP7 high expression phenotype (listed in Table S2). The main results showed that the terms cytokine-cytokine receptor interaction (Figure 6A); calcium signaling pathway (Figure 6B); vascular smooth muscle contraction (Figure 6C); cell adhesion related terms such as ECM receptor interaction (Figure 6D), cell adhesion molecules cams (Figure 6E) and focal adhesion (Figure 6F); and immune cell-related terms such as hematopoietic cell lineage (Figure 6G), complement coagulation cascades (Figure 6H) and leukocyte transendothelial migration (Figure 6I) were significantly enriched. Other enriched terms, such as Hedgehog signaling pathway, gap junction, regulation of actin cytoskeleton, MAPK signaling pathway, JAK-STAT signaling pathway are listed in Table S2.

### IGFBP7 expression correlated with the infiltration levels of immune cells in GC

As the enrichment analysis above indicated that cytokine-cytokine receptor interactions and immune cell related pathways were noticeably enriched, we wondered that whether immune cell infiltration was involved in the pathogenic role of IGFBP7 in GC. Therefore, we explored the relationship between IGFBP7 expression and infiltrating immune cells in GC using the TIMER database. The results showed that high IGFBP7 expression had the most significant correlation with macrophages (Cor=0.696, *P*=6.97e-55), followed by dendritic cells (Cor =0.494, *P*=3.43e-24), CD4+ T cells (Cor =0.433, *P*=3.66e-18), CD8+ T cells (Cor=0.312, *P*=8.37e-10) and neutrophils (Cor=0.301, *P*=3.50e-09) (Figure 6).

In addition, we also analyzed the correlations between IGFBP7 expression and immune markers of tumor associated macrophages (TAMs), M1 macrophages, dendritic cells, T cells and neutrophils. The results revealed that IGFBP7 expression had a strong correlation with gene markers of TAMs, DCs, Tregs, Th1, Th2 cells and neutrophils (Table 3). However, the expression of *NOS2*, *IRF5, PTGS2* and *CD68* in M1 macrophages showed a relatively weak correlation with IGFBP7 expression (Table 3). Overall, the results demonstrated that IGFBP7 had a strong relationship with immune cell infiltration in GC.

## Discussion

The current findings reveal that the mRNA expression of IGFBP7 is divergent in different cancers, which suggests that IGFBP7 functions as both an oncogene or tumor suppressor according to the cancer types. Using large transcriptome sequencing data, we demonstrated that IGFBP7 mRNA was overexpressed in GC specimens and correlated with stage, grade, tumor status and *H. pylori* infection. Furthermore, we found that a high IGFBP7 expression level was significantly associated with poor survival in GC, and served as an independent risk factor for GC. A previous study based on immunohistochemical staining and mRNA revealed that IGFBP7 was positively correlated with invasion, lymph node metastasis and worse survival in GC 25. Another study based on a high-content antibody microarray found that IGFBP7 exhibited the most profound variation (log2FC=2.04, adj.p-vaule <0.05) between 16 pairs of gastric adenocarcinomas and adjacent tissues and may be a candidate marker for the diagnosis of GC 29 Altogether, these results suggest that IGFBP7 may contribute to the carcinogenic process in GC and serve as a potential prognostic marker.

Increasing evidence has reported that IGFBP7 expression is regulated by epigenetic changes, especially by DNA methylation in various cancers 16, 30-33. In this study, we found a significantly negative correlation between the expression of IGFBP7 and methylation levels in GC. IGFBP7 methylation levels were decreased in GC compared with normal tissues, and hypomethylation of IGFBP7 was associated with unfavorable survival of GC. Intriguingly, a study from Kim et al revealed that IGFBP7 expression was silenced by aberrant methylation in GC cells and GC tissues 34. The difference in the results may be due to different detection methods and sample sizes, as well as the different expression types of IGFBP7 in GC. Importantly, the current study was a comprehensive bioinformatics analysis based on multiple databases and large numbers of GC samples. In addition, IGFBP7 has been identified as a novel tumor stroma marker that expressed in activated tumor-associated fibroblasts (CAFs) in several epithelial tumor types 35. As activated CAFs in carcinomas play an important role in tumor cell growth, invasion and metastasis, contrasting expression of IGFBP7 in tumor microenvironment (TME) cells may account for the divergent results in different studies.

Coexpressed genes usually have similar functions. The coexpression analysis indicated that IGFBP7 was highly positively correlated with the top eight coexpressed genes (*LHFPL6*, *MGP*, *SEPTIN4*, *HSPB2*, *ACTA2*, *LAYN, NDN*, and *GGT5*), and all these genes served as risk factors for unfavorable survival of GC. Some of these genes have been reported to promote tumor progression and poor prognosis in malignancy 36-39. It seems that IGFBP7 and its coexpressed genes may act as good prognosis markers for GC. To explore the potential biological processes of IGFBP7, we conducted GO and KEGG analyses of the coexpressed genes and GSEA analysis for IGFBP7. The enrichment analysis of the top 200 coexpressed genes showed that extracellular matrix (ECM) related processes were significantly enriched. The GSEA revealed that the terms cytokine-cytokine receptor interaction, ECM-related, adhesion receptor interaction, cell adhesion molecules cams, focal adhesion, and immune cell related pathways were significantly enriched in IGFBP7 high expression phenotype. It has been reported that ECM components such as collagen, fibroblasts and their associated signaling molecules contribute to tumor cell proliferation, migration and invasion in various cancers 40, 41. Moreover, IGFBP7 has been reported to regulate epithelial-mesenchylmal transition (EMT) in some solid tumors 12, 42, 43. The above results suggest that IGFBP7 and its coexpressed genes may be involved in ECM-related pathways, which would contribute to the utility of IGFBP7 to predict poor prognosis of GC. *H. pylori* infection is well known to cause chronic gastric inflammation and eventually GC. EMT and the tumor microenvironment contribute to *H. pylori* induced GC 44. In this study, we demonstrated that IGFBP7 was highly expressed in GC samples with *H. pylori* infection. As a secret protein, IGFBP7 contributed significantly to immune modulation of mosenchymal stromal cells in experimental colitis and was involved in the modulation of cytokine production by T cells 45. Therefore, IGFBP7 may be involved in *H. pylori* related inflammatory response during the progression of GC. Other enriched pathways related to high IGFBP7 expression, such as the Hedgehog signaling pathway, MAPK signaling pathway and JAK-STAT signaling pathway have been widely reported to contribute to the carcinogenic process of GC 46-48. Hence, IGFBP7 might participate in a variety of biological pathways in the carcinogenic process of GC.

Tumor-infiltrating immune cells are important components of the tumor stroma and contribute to tumor progression and response to cancer therapy 49. Our results demonstrated that there is a robustly positive relationship between IGFBP7 expression level and infiltration of macrophages, DCs, CD4+ T cells and CD8+ T cells, which indicated that IGFBP7 may be involved in regulating tumor immunology in GC. IGFBP7 expression showed a strong correlation with TAM-related gene markers but a weak correlation with M1-related gene markers. TAM infiltration has been reported to be associated with various malignant phenotypes and poor prognosis in GC 50, 51. These above results suggested the hypothetical role of IGFBP7 in regulating the TAM phenotype in GC, which plays an important role in gastric carcinogenesis and prognosis. In addition, a relatively strong correlation between IGFBP7 expression and gene markers of DCs, Treg, Th1 and Th2 cells indicated the potential role of IGFBP7 in regulating DCs and T cell function in GC. In response to *H. pylori* infection in the gastric mucosa, various immune cells including macrophages, DCs and Th1 polarized immune cells are activated and recruited to gastric epithelial cells by a variety of bacterial factors 52. The results indicate that IGFBP7 may play an important role in the immune regulation of *H. pylori-*related gastric diseases. In fact, some members of the IGFBP superfamily such as IGFBP1, IGFBP2 and IGFBP5 have been reported to be upregulated by *H. pylori* infection 53-55. Whether IGFBP7 participates in the pathogenesis of *H. pylori*-related diseases requires further experimental verification.

## Conclusion

In conclusion, we demonstrated that increased IGFBP7 expression correlates with poor prognosis and immune infiltration in GC. Our work highlights the important role of IGFBP7 on the carcinogenicity and prognosis in GC.

## Supplementary Material

Supplementary figures.

Supplementary tables.

## Figures and Tables

**Figure 7:**
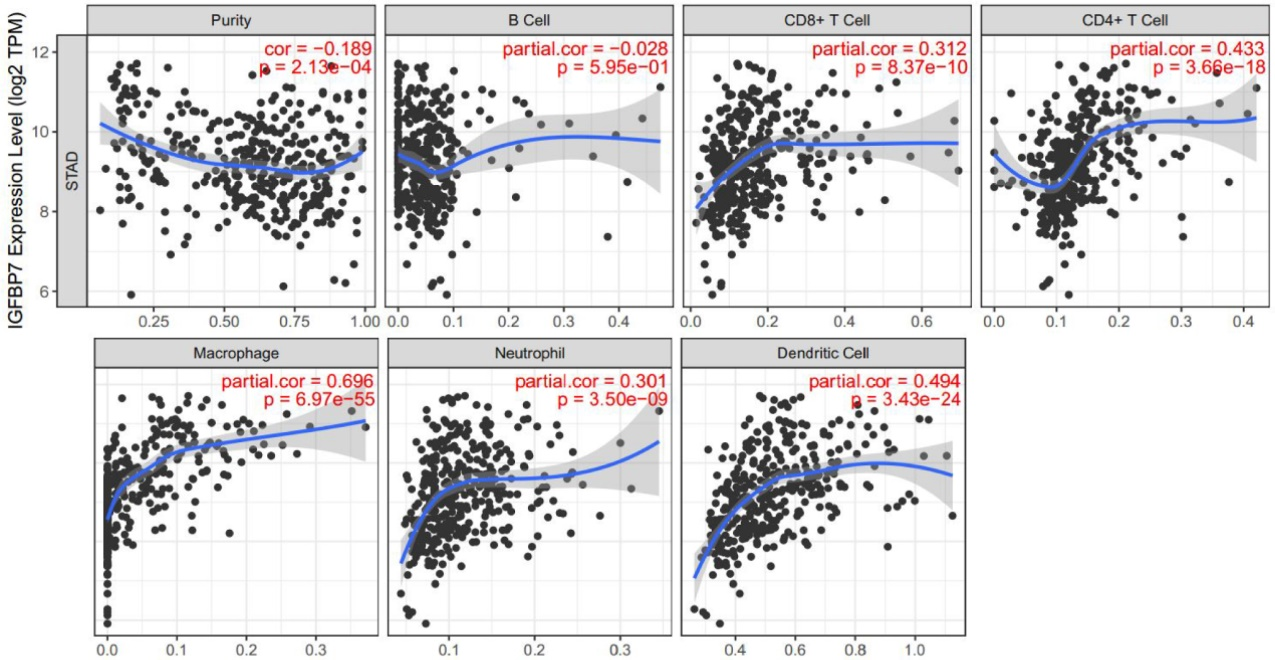
Correlation of IGFBP7 expression with immune cell infiltration in GC. The study was performed in the TIMER database. Spearman's correlation was used to evaluate the correlation between IGFBP7 expression and immune cells.

**Table 1:** The mRNA expression of IGFBP7 was upregulated in different types of gastric cancer compared with normal gastric tissues

Different types of GC vs. Normal	Fold Change	t-test	*P*-value	Reference
Diffuse Gastric Adenocarcinoma	4.217	14.986	6.31E-13	Chen Gastric
Gastric Instinal type Adenocarcinoma	2.333	11.245	6.19E-19	Chen Gastric
Gastric Mixed Adenocarcinoma	4.414	8.377	1.24E-05	Chen Gastric
Gastric Instinal type Adenocarcinoma	2.721	7.102	3.26E-09	DErrico Gastric
Gastric Mixed Adenocarcinoma	4.669	7.154	1.54E-06	DErrico Gastric
Diffuse Gastric Adenocarcinoma	2.238	4.998	4.16E-06	Cho Gastric
Gastric Adenocarcinoma	2.139	2.529	3.50E-02	Cho Gastric
Gastric Instinal type Adenocarcinoma	1.987	3.061	3.00E-03	Cho Gastric

**Table 2:** Univariate and multivariate analysis of IGFBP7 mRNA levels and clinical parameters in TCGA gastric cancer patients

Variables	Univariate analysis			Multivariate analysis		
	HR	95%CI	*P* value	HR	95%CI	*P* value
**Age (years)**						
>60 vs ≤60	1.68	1.13-2.49	0.010**	1.75	1.18-2.59	0.006**
**Gender**						
Male vs Female	1.47	1-2.15	0.049*	1.44	0.98-2.11	0.06
**Stage**						
III+IV vs I+II	1.72	1.19-2.48	0.004**	1.48	0.90-2.43	0.12
**Grade**						
G3 vs G1+G2	1.28	0.089-1.85	0.186	NA	NA	NA
**T stage**						
T3+T4 vs T1+T2	1.46	0.94-2.26	0.096	NA	NA	NA
**N stage**						
N1~N3 vs N0	1.70	1.11-2.61	0.014*	1.29	0.72-2.87	0.39
**M stage**						
M1 vs M0	1.63	0.85-3.11	0.141	NA	NA	NA
**IGFBP7**						
High vs Low	1.5	1-2.1	0.032*	1.51	1.06-2.15	0.022*

*P<0.05; **P<0.01.

**Table 3:** Correlation analysis between IGFBP7 mRNA expression and gene markers of immune cells in gastric cancer

Immune cells	Gene marker	Correlation coefficient	*P* value
TAM (M2)	CCL2	0.597	0
	IL10	0.402	1.53E-17
	CD206	0.392	0
	CD163	0.41	4.52E-35
	VSIG4	0.488	5.44E-51
	CSF1R	0.564	0
	FCGR2A	0.453	0
M1	NOS2	-0.067	1.73E-01
	IRF5	0.255	1.54E-07
	PTGS2	0.191	8.93E-05
	CD68	0.238	1.06E-06
DCs	ITGAX	0.4	0
	CD1C	0.511	5.88E-29
	NRP1	0.661	0
	THBD	0.587	9.92E-40
Treg	FOXP3	0.345	6.91E-13
	STAT5B	0.455	0
	TGFB1	0.611	8.77E-44
Th1	STAT4	0.358	6.97E-14
	TBX21	0.303	3.07E-10
	CD4	0.494	0
	TNF	0.101	3.98E-02
Th2	GATA3	0.427	0
	CXCR4	0.504	4.58E-28
	CCR8	0.39	1.45E-16
	STAT5A	0.336	1.93E-12
Neutrophils	MPO	0.355	8.92E-14
	ITGAM	0.449	0
	CCR7	0.438	0
	CD16 (FCGR3A)	0.372	4.81E-15
	CD32 (FCG2A)	0.453	0

## Abbreviations

IGFBP7: insulin-like growth factor binding protein-7
GC: gastric cancer
TIMER: Tumor Immune Estimation Resource
TCGA: The Cancer Genome Atlas
GEO: Gene Expression Omnibus
GEPIA: Gene Expression Profiling Interactive Analysis
ECM: extracellular matrix
GSEA: gene set enrichment analysis
*H. pylori*: Helicobacter pylori
IGF: insulin-like growth factor
IGFBPs: IGF binding proteins
NES: normalized enrichment score
FDR: false discovery rate
STAD: stomach adenocarcinoma
*LHFPL6*: tetraspanin subfamily member 6
*MGP*: matrix Gla protein
*SEPTIN4*: Septin4
*HSPB2*: heat shock protein family B (Small) member 2
*ACTA2*: actin alpha 2, smooth muscle
*LAYN*: layilin
*NDN*: necdin, MAGE family member
*GGT5*: gamma-glutamyltransferase 5
GO: gene ontology
BP: biological process
CC: cellular component
MF: molecular function
TAMs: tumor associated macrophages
OS: overall survival
FPS: first progression survival
PPS: post progression survival
