# Supplementary material for: Increased IGFBP7 Expression Correlates with Poor Prognosis and Immune Infiltration in Gastric Cancer
Source: J Cancer. 2021 Jan 1;12(5):1343–55. doi: 10.7150/jca.50370 (PMC7847654; doi:10.7150/jca.50370)
Supplement: Supplementary file 1 — Supplementary figures. [file jcav12p1343s1.pdf]

Supplementary information

Increased IGFBP7 Expression Correlates with Poor Prognosis and Immune Infiltration in Gastric Cancer: An Integrated Boinformatics Analysis

Qiaoyun Zhao1, Rulin Zhao1, Conghua Song1, Huan Wang1, Jianfang Rong1, Fangfei Wang1, Lili Yan2, Yanping Song1, Yong Xie1

Additional file 1: Table S1. GEO datasets used in the study.

Table S2. Significant KEGG terms in GSEA analysis of IGFBP7. NES: normalized enrichment score; NOM p-val: nominal p val; FDR q-val: false discovery rate. NES>1&FDR q-val <0.05 was considered statistically significant.

Additional file 2: Figure S1. IGFBP7 mRNA expression and methylation status in gastric cancer. A, IGFBP7 mRNA expression and methylation status in GC data as analyzed in MEXPRESS. The samples are ordered according to IGFBP7 expression from the lowest (left) to the highest (right). The bottom of the figure shows the 450K probes related to the IGFBP7 gene. The probes in the promoter region of IGFBP7 are highlighted with a black line. * P< 0.05. ** P < 0.01, *** P< 0.001. B. Promoter methylation level of IGFBP7 as assessed with UALCAN. C. The relationship between promoter methylation of IGFBP7 and overall survival as assessed with the Xena database.

Figure S2. Gene ontology and pathway enrichment analyses of the top 200 coexpressed genes of IGFBP7. Briefly, the top 200 coexpressed genes according to the adjusted p value in cBioPortal database analysis were used, and enrichment analysis was performed using clusterProfiler package in R. A-C, Gene ontology (GO) analysis for BP (A), CC (B) and MF (C). BP, biological processes, CC, cellular component, MF, molecular function. D. KEGG pathways analysis of top 200 coexpressed genes.

Table S1. GEO datasets used in the study.

Accession ID	Platform	Gastric cancer(N)	Normal tissues(N)	Paired	Clinical data	Years	Reference
GSE54129	GPL570	111	21	No	No	2017	Liu et al
GSE79973	GPL570	10	10	Yes	No	2016	He et al
GSE118916	GPL15207	15	15	Yes	No	2019	Li et al
GSE15459	GPL570	198	0	No	Yes	2009	Ooi et al

Table S2. Significant KEGG terms in GSEA analysis of IGFBP7.

NAME	NES	NOM p-val	FDR q-val	FWER p-val	RANK
KEGG_NEUROACTIVE_LIGAND_RECEPTOR_INTERACTION	2.425902	0	0	0	6878
KEGG_CYTOKINE_CYTOKINE_RECEPTOR_INTERACTION	2.302273	0	0.00073	0.002	8219
KEGG_VASCULAR_SMOOTH_MUSCLE_CONTRACTION	2.279336	0	0.00133	0.004	3561
KEGG_CALCIUM_SIGNALING_PATHWAY	2.239484	0	0.00179	0.007	5038
KEGG_ECM_RECEPTOR_INTERACTION	2.23756	0.002008	0.00143	0.007	4462
KEGG_DILATED_CARDIOMYOPATHY	2.221198	0	0.00164	0.01	4462
KEGG_FOCAL_ADHESION	2.190292	0	0.0017	0.012	5001
KEGG_HEDGEHOG_SIGNALING_PATHWAY	2.175366	0	0.00164	0.014	3596
KEGG_HYPERTROPHIC_CARDIOMYOPATHY_HCM	2.162431	0	0.00178	0.017	4462
KEGG_HEMATOPOIETIC_CELL_LINEAGE	2.138025	0.001919	0.00209	0.02	7305
KEGG_CELL_ADHESION_MOLECULES_CAMS	2.122281	0	0.00277	0.029	6143
KEGG_GAP_JUNCTION	2.069905	0	0.00466	0.042	2766
KEGG_BASAL_CELL_CARCINOMA	2.051412	0	0.00531	0.049	3596
KEGG_COMPLEMENT_AND_COAGULATION_CASCADES	2.031948	0.001992	0.00626	0.061	4482
KEGG_GLYCOSAMINOGLYCAN_BIOSYNTHESIS_CHONDROITIN_SULFATE	1.954636	0.007576	0.01608	0.135	7100
KEGG_REGULATION_OF_ACTIN_CYTOSKELETON	1.952644	0.005964	0.01571	0.139	4875
KEGG_MELANOMA	1.948851	0.003891	0.01525	0.142	3260
KEGG_LEUKOCYTE_TRANSENDOTHELIAL_MIGRATION	1.900294	0.00396	0.02389	0.188	8337
KEGG_MELANOGENESIS	1.883248	0	0.0263	0.218	6152
KEGG_MAPK_SIGNALING_PATHWAY	1.868716	0.003906	0.02864	0.241	7597
KEGG_ARRHYTHMOGENIC_RIGHT_VENTRICULAR_CARDIOMYOPATHY_ARVC	1.858917	0.009862	0.03097	0.263	4462
KEGG_JAK_STAT_SIGNALING_PATHWAY	1.804173	0.00823	0.04355	0.338	8481

NES: normalized enrichment score; NOM p-val: nominal p val; FDR q-val: false discovery rate. NES>1&FDR q-val <0.05 was considered statistically significant.

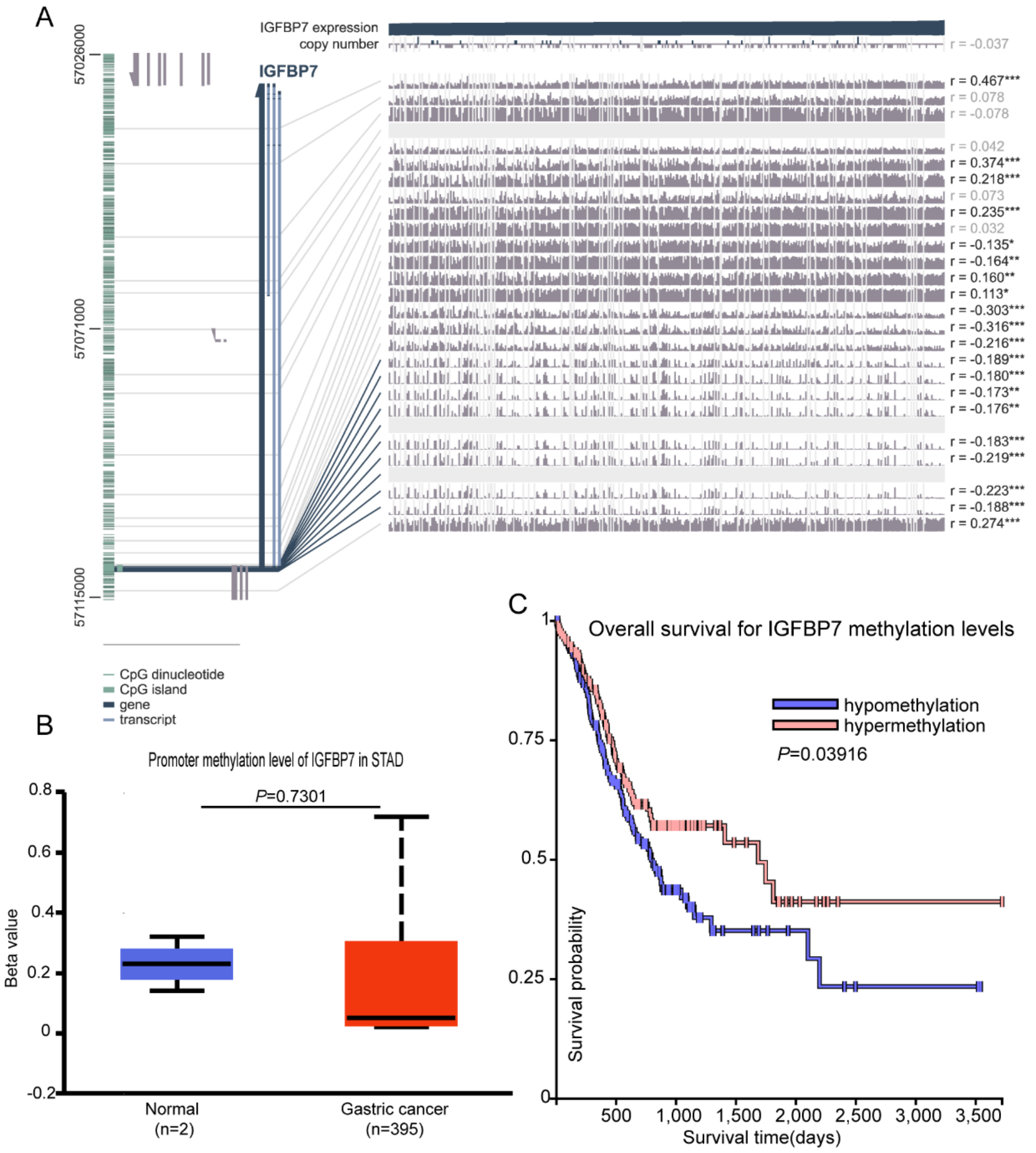

Figure S1. IGFBP7 mRNA expression and methylation status in gastric cancer. A, IGFBP7 mRNA expression and methylation status in GC data as analyzed in MEXPRESS. The samples are ordered according to IGFBP7 expression from the lowest (left) to the highest (right). The bottom of the figure shows the 450K probes related to the IGFBP7 gene. The probes in the promoter region of IGFBP7 are highlighted with a black line. * P< 0.05. ** P < 0.01, *** P< 0.001. B. Promoter methylation level of IGFBP7 as assessed with UALCAN. C. The relationship between promoter methylation of IGFBP7 and overall survival as assessed with the Xena database.

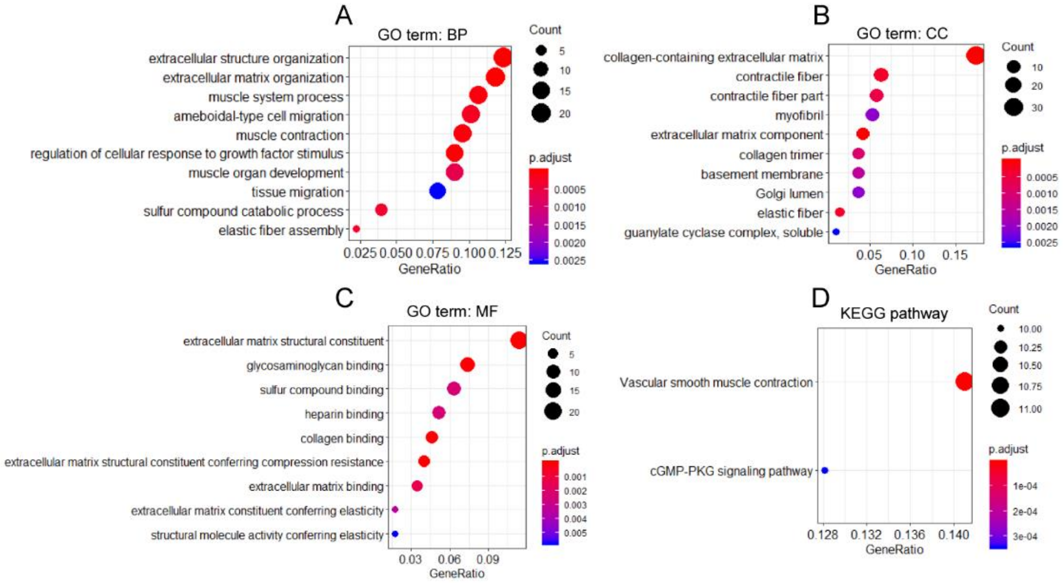

Figure S2. Gene ontology and pathway enrichment analyses of the top 200 coexpressed genes of IGFBP7. Briefly, the top 200 coexpressed genes according to the adjusted p value in cBioPortal database analysis were used, and enrichment analysis was performed using clusterProfiler package in R. A-C, Gene ontology (GO) analysis for BP (A), CC (B) and MF (C). BP, biological processes, CC, cellular component, MF, molecular function. D. KEGG pathways analysis of top 200 coexpressed genes.
